# Bone health: an important consideration in coeliac disease 

**Published:** 2020

**Authors:** William Shanahan, Jayne Shanahan

**Affiliations:** *Department of Medicine, University Hospital Waterford, Dunmore Road, Co. Waterford, X91ER8E, Ireland*

**To The Editor**

Shiha et al pose a contentious question in their recent article entitled “Diagnosing coeliac disease in the elderly: a United Kingdom cohort study” (1). They muse at the necessity for active case finding of coeliac disease in the elderly, implying that the burden of breaking nutritional habits may exceed the benefits of diagnosis. 

While one may concede that the adage “scientia potentia est” may not always hold true, we are inclined to disagree with the authors in this instance. Bone health is an important consideration both in the elderly and in those with coeliac disease, independently. It follows that this should be of particular concern with respect to the elderly with coeliac disease. Regarding the authors reference to the need to establish the risks of not pursuing a diagnosis, we feel their suggestion belies a lack of appreciation for the potential to recognise and prevent bone related morbidity in such patients. An audit of patients within the catchment of our tertiary level hospital in the South-East of Ireland shows that such an under-appreciation may be widespread. The British Society of Gastroenterology guidelines on coeliac disease state that patients should undergo a vitamin D level check at diagnosis (2). On reviewing our laboratory records of patients with a positive IgA anti-tissue transglutaminase for over 18 months, from January 2018 to June 2019, we found that only 14.6% of patients had their vitamin D levels checked within 6 months of a first positive result.

Coeliac disease is associated with an increased risk of osteoporosis and fracture(3), and just 1 year of adhering to a gluten free diet in such patients has been shown to lead to an improvement in objective markers of bone mineral density(4). In light of this, we feel the need to express our respectful disagreement with the proposal to not seek out a diagnosis of coeliac disease in old age.Additionally, we should be reminding our clinician colleagues of its relationship with bone disease and to actively manage those patients who have multiple osteoporotic risk factors. This appears to be especially true in the case of coeliac disease given the poor compliance with guidelines in relation to vitamin D assessment in the cohort of patients within the catchment of our institution. 

## Conflict of interests

The authors declare that they have no conflict of interest.
